# The relationship between the femoral artery and vastus medialis muscle coverage at the adductor hiatus during medial closed wedge distal femoral osteotomy in valgus knees

**DOI:** 10.1002/jeo2.12082

**Published:** 2024-07-15

**Authors:** Fumiyoshi Kawashima, Hiroshi Takagi, Koji Kanzaki

**Affiliations:** ^1^ Department of Orthopedic Surgery Showa University Fujigaoka Hospital Yokohama Japan

**Keywords:** adductor hiatus, femoral artery, medial closed‐wedge distal femoral varus osteotomy, muscle morphology, popliteus artery, vastus medialis

## Abstract

**Purpose:**

The purpose of this study was to examine the location where the femoral artery contacts the vastus medialis at the adductor tendon hiatus, which is important when using the subvastus approach in medial closed wedge distal femoral osteotomy. We evaluated the correlation between differences in height, vastus medialis morphology, and lower limb alignment.

**Methods:**

Sixty knees (16 male, 44 female) that underwent plain computer tomography (CT) were included. Using the radiographic hip‐knee‐ankle (HKA) angle as a reference, the knees were divided into three groups of 20 knees: valgus, varus, and neutral. The mechanical lateral distal femoral angle (mLDFA) and distance from the medial femoral epicondyle to the centre of the femoral head (D1) were measured on full‐length weight‐bearing anteroposterior radiographs. The first cross‐section on CT where the vastus medialis muscle and femoral artery connect was defined as the cross‐sectional image for measurement. The direct distance from the medial epicondyle to the measured cross‐sectional image (D2) was measured in the coronal view. The ratio of the vastus medialis muscle width to the femoral posterior wall width was defined as the vastus medialis muscle coverage ratio (CR). Correlations between each measurement and group were evaluated.

**Results:**

There was a positive correlation between D1 and D2 in the overall, neutral, and varus groups; however, there was no correlation in the valgus group. A positive correlation was observed in terms of the relationship between CR and D2 in the overall and valgus groups. In addition, there was no statistically significant difference in the correlation between the mLDFA and D2, with patient height as a control variable overall and in all groups.

**Conclusion:**

In the valgus group, distance to the adductor hiatus was correlated with vastus medialis coverage. Overhang of the vastus medialis may be an important influencing factor of femoral and popliteus artery position.

**Level of Evidence:**

Level III, retrospective cohort study.

**Abbreviations**
ACLR: anterior cruciate ligament reconstruction
CR: vastus medialis muscle coverage ratio
D1: distance from the medial femoral epicondyle to the centre of the femoral head
D2: direct distance from the medial epicondyle to the measured cross‐sectional image
HKA: hip‐knee‐ankle
HTO: high tibial osteotomy
KL classification: Kellgren‐Lawrence classification
MCWDFO: medial closed‐wedge distal femoral varus osteotomy
MIPO: minimally invasive plate osteosynthesis
mLDFA: mechanical lateral distal femoral angle
OA: osteoarthritis
TKA: total knee arthroplasty
UKA: unicompartmental arthroplasty

## INTRODUCTION

**Figure 1:**
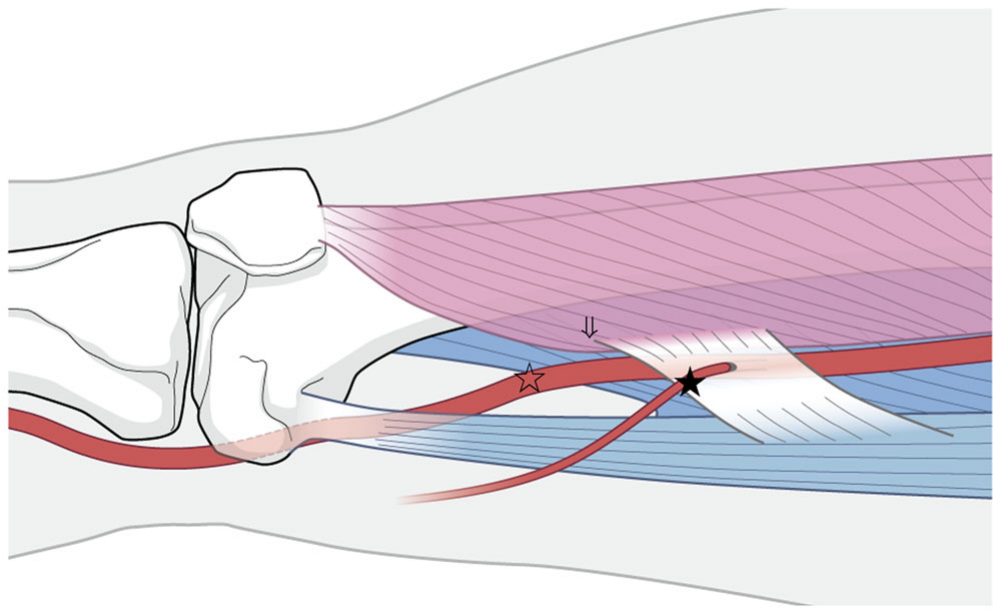
Transverse image of the distal femur on plain computer tomography. When considering the cross‐section of the distal femur, the lateral cortical surface has a steep overall slope with respect to the posterior cortical surface of the femur (curly bracket); however, the angle of the slope of the medial cortical surface changes significantly in the posterior and anterior sections, and the anterior exhibits a mild slope (solid arrow, outlined arrow). Therefore, when the medial surface of the femur is viewed from the mediolateral side during surgery in the supine position, there are cases in which the anterior and posterior border of the medial surface and the anteroposterior width are difficult to assess.

Medial closed‐wedge distal femoral varus osteotomy (MCWDFO) is a useful surgical technique for treating valgus knee osteoarthritis (OA) originating from the distal femur [3, 8], and the subvastus approach is commonly used to expose the surgical field in these cases [14]. With the adductor hiatus as the border, the popliteal artery runs posterior to the femur, translates medially and anteriorly, enters the adductor canal, and becomes the femoral artery. Considering the potential risk of vascular injury via the subvastus approach, it is particularly important to consider the potential damage to the femoral artery when performing MCWDFO [2]. At a position proximal to the adductor hiatus where the artery meets the posterior border of the vastus medialis muscle and extends into the common femoral artery, care must be taken when exposing the surgical field and fixing proximal screws with the plate (Figure 1).

There are many previous reports on the distance from the distal femur or border of the patella to the adductor hiatus [5, 11, 15, 17, 20]. However, there are no reports in the literature on statistical differences in this distance due to lower limb alignment. MCWDFO is a procedure for the valgus knee, and an evaluation of characteristics regarding the course of the popliteal fossa and femoral artery through the adductor hiatus in the valgus knee would be beneficial for surgical decision making. The purpose of this study was to use plain computer tomography (CT) to examine the position where the adductor hiatus meets the vastus medialis muscle with a focus on the differences and correlations among patient height, vastus medialis muscle morphology, and lower limb alignment. In addition, we aimed to evaluate the safety of proximal surgical exposure using the subvastus approach. We hypothesized that the distance from the distal femur to the adductor hiatus is proportional to patient height and vastus medialis muscle coverage.

## METHODS

Patients who underwent plain CT for preoperative planning between January 2016 and December 2019 at our hospital were included. CT images obtained with a 64‐row‐detector CT scanner (Discovery CT 750HD, GE Healthcare) were evaluated. Among 140 patients who underwent whole‐leg CT scans for preoperative planning at our hospital, 60 patients (16 males and 44 females) with no history of ipsilateral knee surgery and a BMI of less than 25 were included in the study. The following patients were excluded: those with inflammatory diseases such as rheumatoid arthritis, condylar necrosis, the severest grade 4 varus OA as classified by the Kellgren–Lawrence (KL) classification of OA [10] and hip‐knee‐ankle (HKA) of 15° or greater, a flexion contracture of 10° or greater, a history of ipsilateral lower extremity fracture, a history of knee surgery for meniscal or cartilage damage, OA changes in the ipsilateral hip joint, acetabular dysplasia, and a history of lower limb surgery. All patients underwent plain radiography, and images of the whole lower limb of both legs were taken in the weight‐bearing position. This research was approved by the IRB of the authors' affiliated institutions. The patients and/or their families were informed that the data from the research would be submitted for publication, and they gave their consent.

Based on a report by Thienpont et al. [21] and the CPAK classification [12], the HKA (varus expressed as negative value) on full‐length standing radiography was divided into three groups of 20 knees: the varus group (HKA less than −2° to less than −15°; CPAK classification of Ⅰ, Ⅳ, Ⅶ), valgus group (greater than 2°; CPAK classification of Ⅲ, Ⅵ, Ⅸ), and neutral group (greater than −2° to less than 2°; CPAK classification of Ⅱ, Ⅴ, Ⅷ). The valgus group had an additional requirement of a mechanical lateral distal femoral angle (mLDFA:°) of 85° or less and valgus originating from the distal femur. In the varus group, there were 10 knees before high tibial osteotomy (HTO), eight knees before total knee arthroplasty (TKA), and two knees before unicompartmental arthroplasty (UKA). The severity of OA on anteroposterior radiography according to the KL classification was as follows: two knees were grade 1, 13 were grade 2, and 5 knees were grade 3. All patients in the neutral group underwent imaging before anterior cruciate ligament reconstruction (ACLR), and all patients in the valgus group underwent imaging prior to MCWDFO.

The mean age was 56.6 ± 21.9 years overall, 31.9 ± 15.9 in the neutral group, 59.6 ± 13.8 in the valgus group, and 74.1 ± 9.2 in the varus group. Because there was a specificity in the age at which the surgery was performed between groups, a significant difference was observed among the three groups. The patient height was 158.5 ± 10.8 overall, 166.1 ± 10.7 in the neutral group, 155.3 ± 6.9 in the valgus group, and 154.5 ± 10.5 in the varus group, with significant differences between the neutral, varus, and valgus groups (Table 1).

**Table 1:** Patient characteristics.

Parameters	Overall	Neutral	Valgus	Varus
Age (years)	55.6 ± 21.9	31.6 ± 15.9	59.6 ± 13.8	74.1 ± 9.2
Patient height (cm)	158.5 ± 10.8	166.1 ± 10.7	155.3 ± 6.9	154.5 ± 10.5

*Note*: Significant difference (*p* < 0.01) was observed in terms of age among all groups: neutral group, valgus group, and varus group. Significant difference was observed in terms of patient height between the neutral group and valgus group, and between the neutral group and varus group.

**Figure 2:**
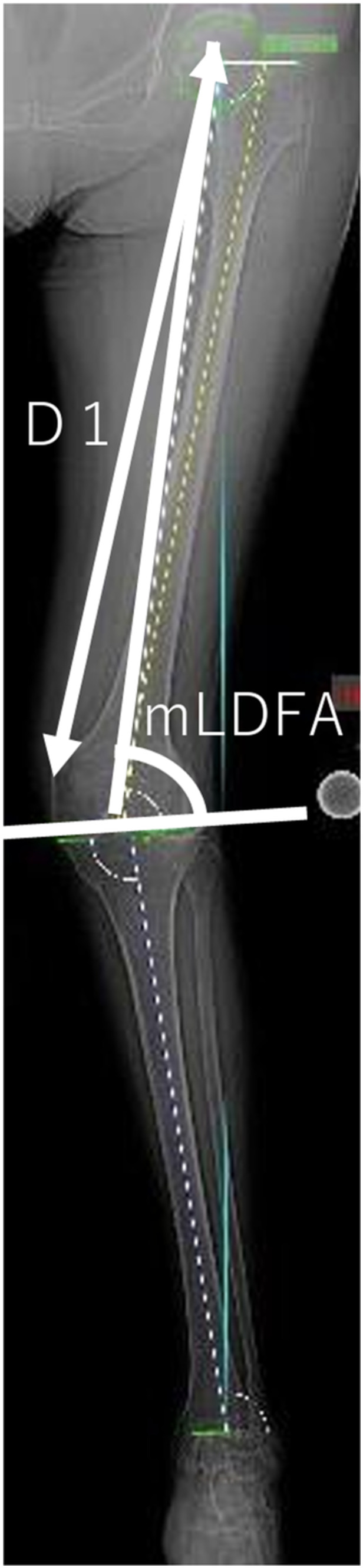
The distance from the medial femoral epicondyle to the centre of the femoral head and the mechanical lateral distal femoral angle (mLDFA) measured in the full‐length anterior‐posterior weight‐bearing view.

**Figure 3:**
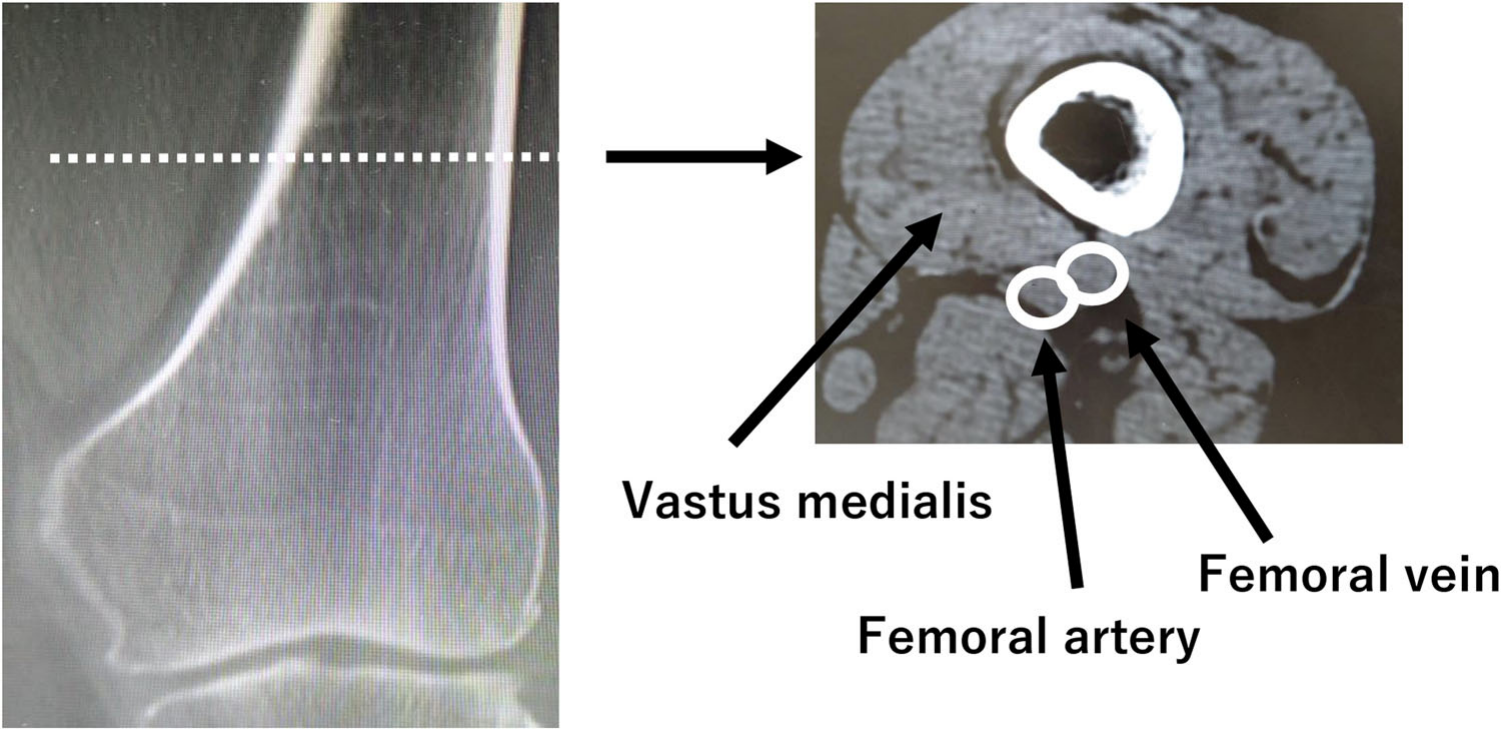
Measurement 1: distance from the medial epicondyle in the coronal plane. The distance (D: mm) from the medial epicondyle in the coronal plane to the cross‐sectional image was measured. Cross‐sectional images were taken perpendicular to the functional axis, and the distance was measured using a line parallel to the functional axis.

**Figure 4:**
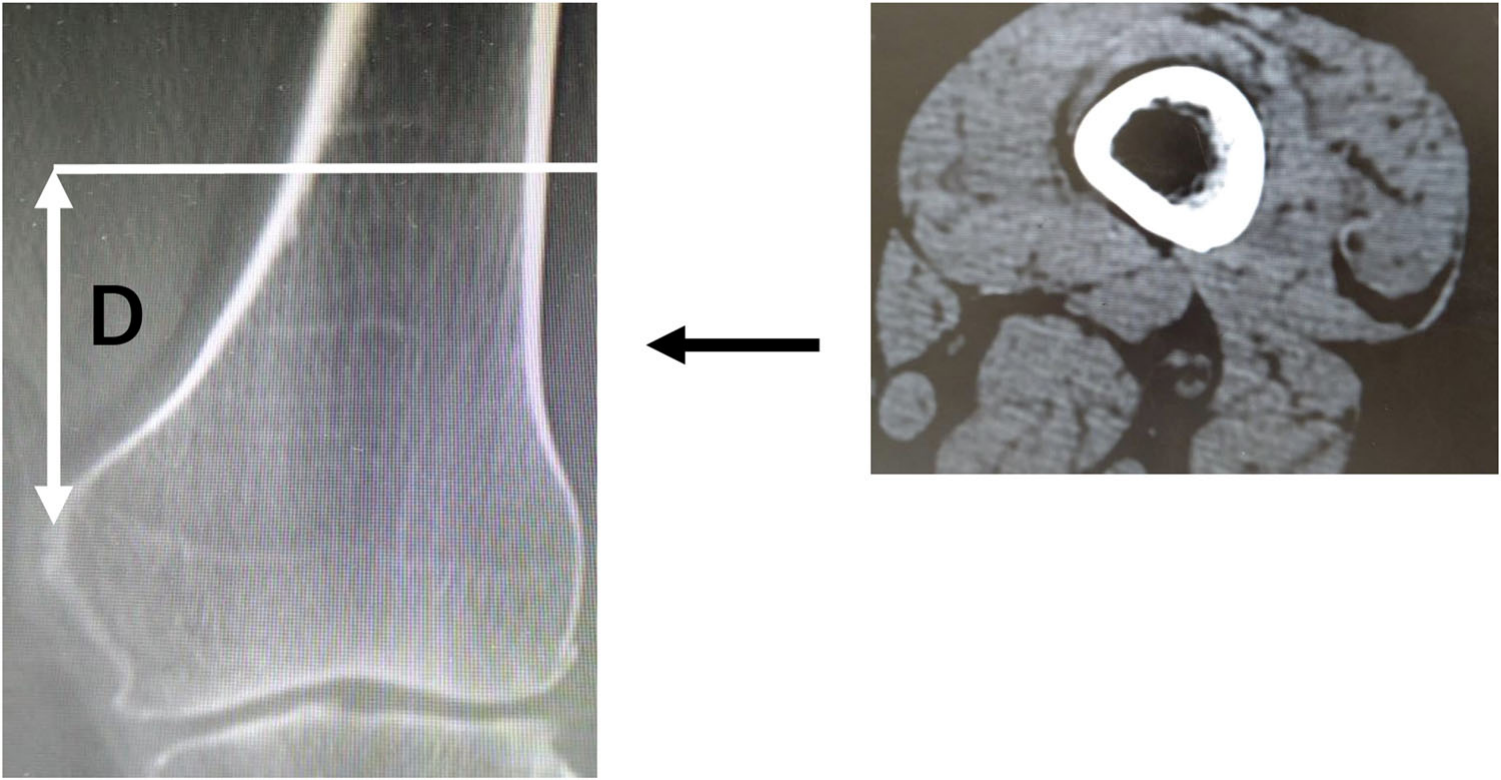
Cross‐sectional plain computed tomography (CT) images for measurement. Full‐length plain CT image of the lower extremity in supine position, with the patella facing forward, 0.65 mm thick, and perpendicular to the functional axis. The first cross‐section on CT where the vastus medialis muscle and popliteal artery distally connect to the adductor hiatus was used.

**Figure 5:**
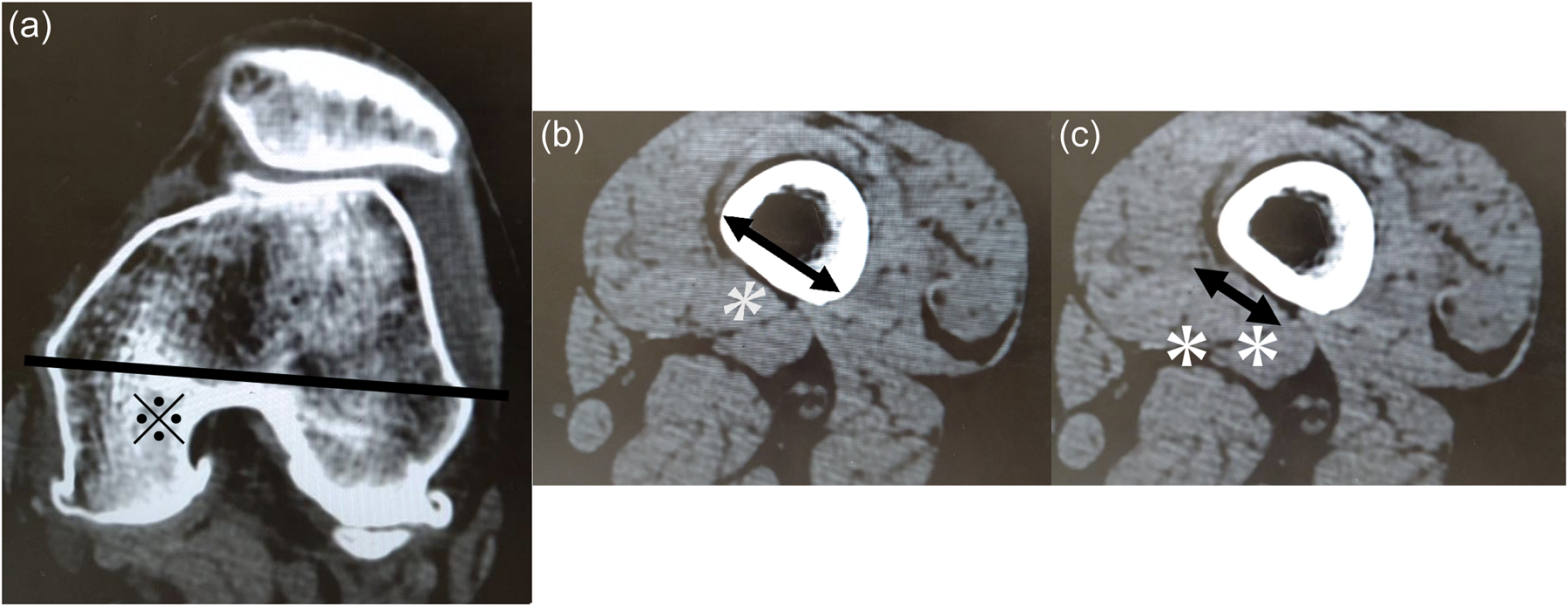
Measurement 2: vastus medialis coverage. The reference mark (※) indicates the epicondylar axis (a). The asterisk (*) indicates the posterior wall width (b). Vastus medialis muscle width (largest‐diameter muscle cross‐section: double asterisk [**]) divided by the posterior wall width was defined as the vastus medialis muscle coverage (%) (c).

For radiographic measurements, CT images were taken with a 64‐row‐detector CT scanner (Discovery CT 750HD, GE Healthcare). The mLDFA was measured to assess distal femoral morphology, and the distance from the medial femoral epicondyle to the centre of the femoral head (D1: mm) was measured in the full‐length anterior‐posterior weight‐bearing view (Figure 2). Next, CT images were taken in a 0.65 mm slice perpendicular to the functional axis in the supine position with the patella facing forward. The direct distance from the medial epicondyle to the slice (D2: mm) was measured using coronal images (Figure 3). Based on a report by Tensho et al. [20], the horizontal cross‐section was defined as the first image obtained from the distal point where the vastus medialis muscle connects to the femoral artery. This cross‐sectional image was chosen because the positions where the adductor hiatus and the vastus medialis begin to connect with the popliteal artery are nearly anatomically identical [5, 6, 11, 17]. There are several methods to determine the optimal cut for proximal oblique osteotomy, which is approximately 4 cm from the medial epicondyle in the coronal plane of the MCWDFO. These include drawing a perpendicular line from the hinge point to the medial cortex and subsequently drawing a bisector of the correction angle from the hinge point with the perpendicular line at the centre [19] or determining the distance from the distal articular surface of the femur [22]. However, these methods tend to produce an osteotomy that is excessively proximal for the East Asian population; therefore, the reference point for distance was set to the medial epicondyle in this study to account for the ethnic makeup of the sample population (Figure 4) [19, 22]. In addition, the epicondylar axis was projected onto the measurement slice, and the distance between the mediolateral bone cortices with the largest diameter was defined as the posterior wall width. The ratio of the vastus medialis width to the posterior wall width was defined as the vastus medialis muscle coverage ratio (CR: %) (Figure 5).

The following measurements were made: D1, D2, mLDFA, overall vastus medialis muscle coverage, mean value of each group and the presence of significant differences between groups. In addition, the correlations between patient height and D1/D2/vastus medialis muscle coverage, vastus medialis muscle coverage and D2, D1, and D2, and mLDFA and D2 with patient height as a control variable were evaluated.

Image measurements were performed by two orthopaedic surgeons using a digital orthopaedics surgical planning tool (medi‐CAD, Hectec) and Synapse (Fuji Film) for plain CT images. To determine the reproducibility of the cross‐sectional CT slice selection, two examiners, including the first author, repeated the CT slide selection process 10 times on separate days for each case. The reproducibility of cross‐sectional CT slice selection was evaluated using the intraclass correlation coefficient. A high correlation was observed between the reliability of one examiner selecting the slice for a case (intraexaminer reliability) of 0.946 and the interexaminer reliability of 0.916 for two examiners selecting the same slice. In this study, measurements were taken using noncontrast‐enhanced images for all cases. The popliteal fossa and femoral artery can be identified without contrast imaging, and there was a high agreement rate between the two examiners; therefore, we determined that there were no problems with the reliability of the diagnosis. Statistical analyses were performed using SPSS statistics ver. 22.0 (IBM Japan Ltd.) analysis software via the Bland‐Altman plot method, Spearman's correlation function, *t*‐test, and partial correlation analysis. The significance level was set to less than 0.05.

## RESULTS

The mean D1 values were 401.2 ± 33.1 (320–488) overall, 414.1 ± 40.4 (350–488) in the neutral group, 397.3 ± 21.2 (374–454) in the valgus group, and 392.9 ± 32.7 (320–485) in the varus group. The mean value was greater in the neutral group than in the varus and valgus groups; however, no statistically significant difference was observed. The mean mLDFA values were 87.9 ± 3.8 (79–96) overall, 88.6 ± 0.7 (88–90) in the neutral group, 84.1 ± 3.6 (79–84) in the valgus group, and 90.7 ± 2.9 (88–96) in the varus group. There were statistically significant differences between the valgus group and the varus group, between the valgus group and the neutral group, and between the varus group and the neutral group. The mean D2 values were 73.6 ± 6.6 (61–88) overall, 74.6 ± 6.4 (64–87) in the neutral group, 74.9 ± 6.4 (65–88) in the valgus group, and 71.5 ± 6.8 (61–83) in the varus group. There were no statistically significant differences overall or between groups. The CRs were 0.42 ± 0.14 (0.14–0.73) overall, 0.43 ± 0.11 (0.17–0.65) in the neutral group, 0.42 ± 0.17 (0.14–0.73) in the valgus group, and 0.41 ± 0.13 (0.17–0.66) in the varus group. No statistically significant differences were observed overall or between groups (Table 2).

**Table 2:** Mean mechanical lateral distal femoral angle (mLDFA), D1, D2, and vastus medialis coverage.

Group	mLDFA	D1 (mm)	D2 (mm)	Vastus medialis coverage (%)
Overall	87.9 ± 3.8	401.2 ± 33.1	73.6 ± 6.6	0.42 ± 0.14
Neutral	88.6 ± 0.7	414.1 ± 40.4	74.6 ± 6.4	0.43 ± 0.11
Valgus	84.1 ± 3.6	397.3 ± 21.2	74.9 ± 6.4	0.42 ± 0.17
Varus	90.7 ± 2.9	392.9 ± 32.7	71.5 ± 6.8	0.41 ± 0.13

*Note*: Statistically significant differences in mLDFA were observed between each group (*p*‐value [Bonferroni correction]: neutral group vs. valgus group, 0.00; neutral group vs. varus group, 0.01; valgus group vs. varus group, 0.00). There were no statistically significant differences between groups in D1, D2, or vastus medialis muscle coverage.

**Figure 6:**
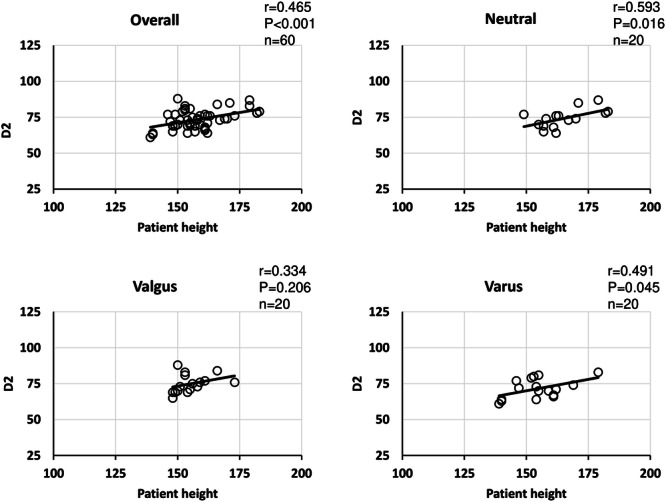
Correlation between height and distance from medial epicondyle (overall). A positive correlation was observed between the overall sample population and the neutral group. Although a weak positive correlation was observed between the varus group, no correlation was observed in the valgus group.

**Figure 7:**
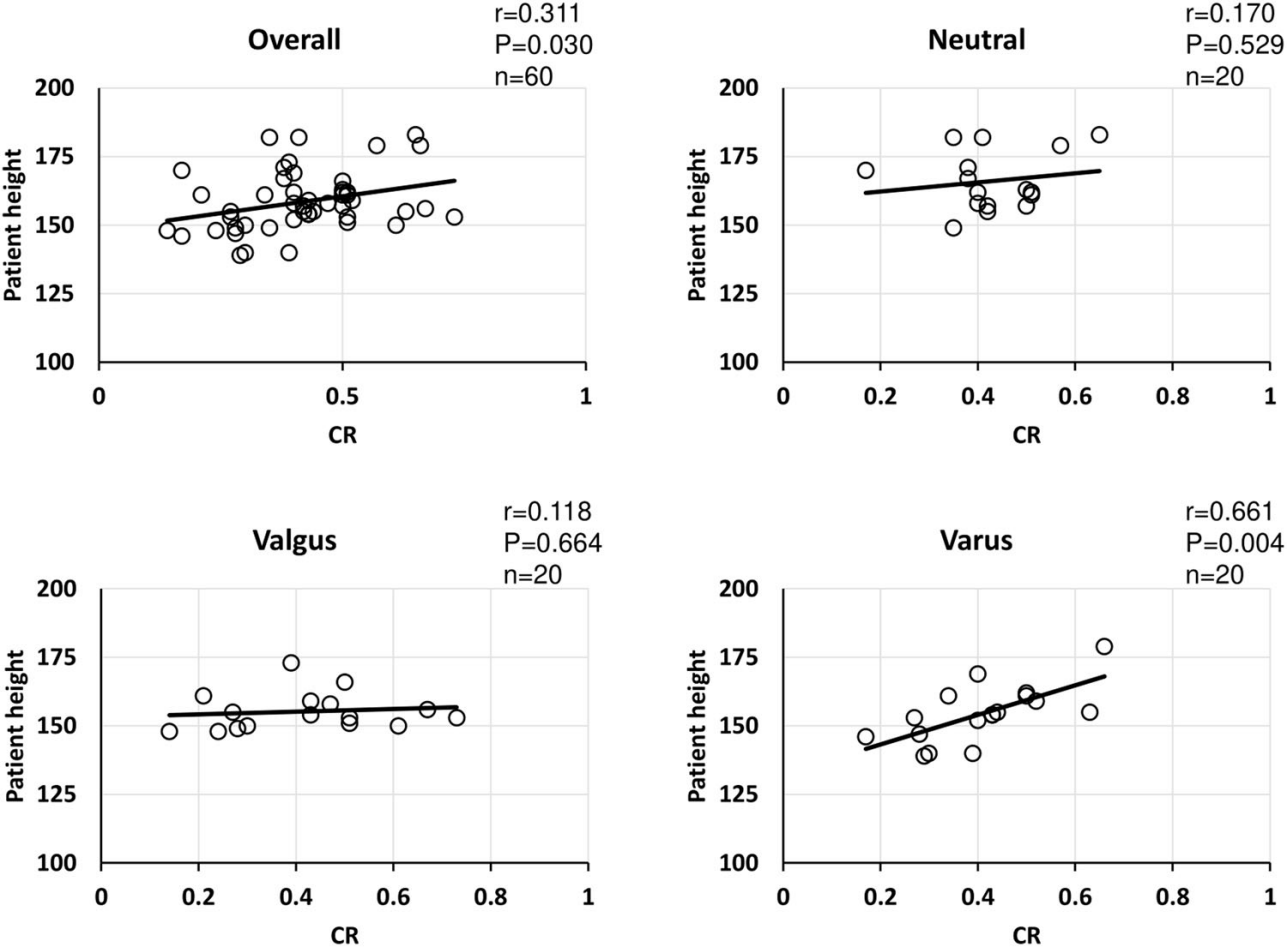
Correlation between patient height and vastus medialis coverage (overall). A weak positive correlation was observed in the overall sample population, and a positive correlation was observed in the varus group alone.

**Figure 8:**
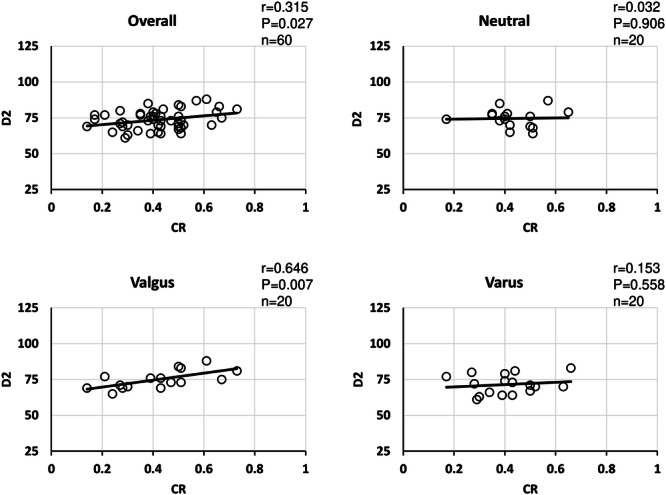
Correlation between vastus medialis coverage and distance from medial epicondyle (overall). A weak positive correlation was observed in the overall sample population, and a positive correlation was observed in the valgus group alone.

**Figure 9:**
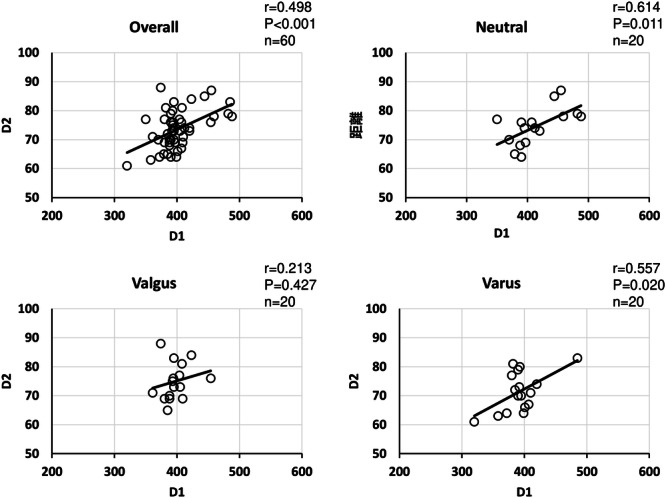
Correlation between D1 and D2. No significant difference was observed in the valgus group alone.

Because there was a significant positive correlation between patient height and D1 overall and in all groups, patient height and femoral length showed a similar positive correlation (Table 3). Regarding the relationship between height and D2, a positive correlation was observed in the overall sample population, in the neutral group, and in the varus group, but not in the valgus group (Figure 6). Regarding the relationship between patient height and CR, a positive correlation was observed in the overall sample population and in the varus/neutral groups. No correlation was observed in the valgus group (Figure 7). Regarding the relationship between CR and D2, a positive correlation was observed in the overall sample population and in the valgus group, but no correlation was observed in the neutral and varus groups (Figure 8). Regarding the relationship between D1 and D2, a positive correlation was observed in the overall sample population, neutral group, and varus group, but no correlation was observed in the valgus group (Figure 9). In addition, the correlations between the mLDFA and D2 with patient height as a control variable were not statistically significantly different overall or in all groups, and there was no significant difference between the severity of varus/valgus deformity of the distal femur and the distance from the medial epicondyle to the adductor hiatus (Table 4).

**Table 3:** Correlation between patient height and D1.

Group	Correlation coefficient	*p*‐Value
Overall	0.897	0.00
Neutral	0.968	0.00
Valgus	0.767	0.001
Varus	0.914	0.00

*Note*: A positive correlation was observed between patient height and D1 overall and within each group. Regardless of alignment, patient height and femoral length were correlated.

**Table 4:** Correlation between mechanical lateral distal femoral angle (mLDFA) and D2 with patient height as a control variable.

Group	Partial correlation coefficient	Significance probability
Overall	−0.120	0.418
Neutral	−0.294	0.288
Valgus	−0.075	0.789
Varus	0.449	0.081

*Note*: Partial correlation analysis excluding the influence of patient height.

## DISCUSSION

Many reports have described a ‘danger zone’ [5, 11, 13, 15, 17], defined as an area in the centre of the femoral shaft where the femoral artery moves from the posterior centre on the distal side to the anteromedial side on the proximal side. However, none have described this zone in terms of lower extremity alignment. Since the anterolateral wall of the adductor canal is the vastus medialis muscle, the position where the femoral artery connects to the lower border of the vastus medialis muscle at the adductor hiatus is believed to be identical [5, 6, 11, 17]. Tensho et al. [20] used plain CT to measure the distance from the superior border of the patella where the vastus medialis muscle and the femoral artery meet. They found that the mean distance was 36 mm (18.6–61.5), and the distance was found to be proportional to patient height and patellar length. In this study, the distance from the medial epicondyle (D2) varied case‐by‐case, with a minimum of 61 mm and a maximum of 88 mm in the overall sample population. However, overall and in the neutral and varus groups, a correlation was observed between patient height and D2. Therefore, similar to the findings of Tensho et al., patient height was one of the important influencing factors for the distance from the distal femur (medial epicondyle) to the adductor hiatus. In the valgus group, however, there was no correlation between the distance to the adductor hiatus, patient height, femoral length, or mLDFA (excluding the effect of patient height), but there was a positive correlation with CR. Therefore, the valgus morphology of the distal femur may have little effect on the position of the adductor hiatus.

**Figure 10:**
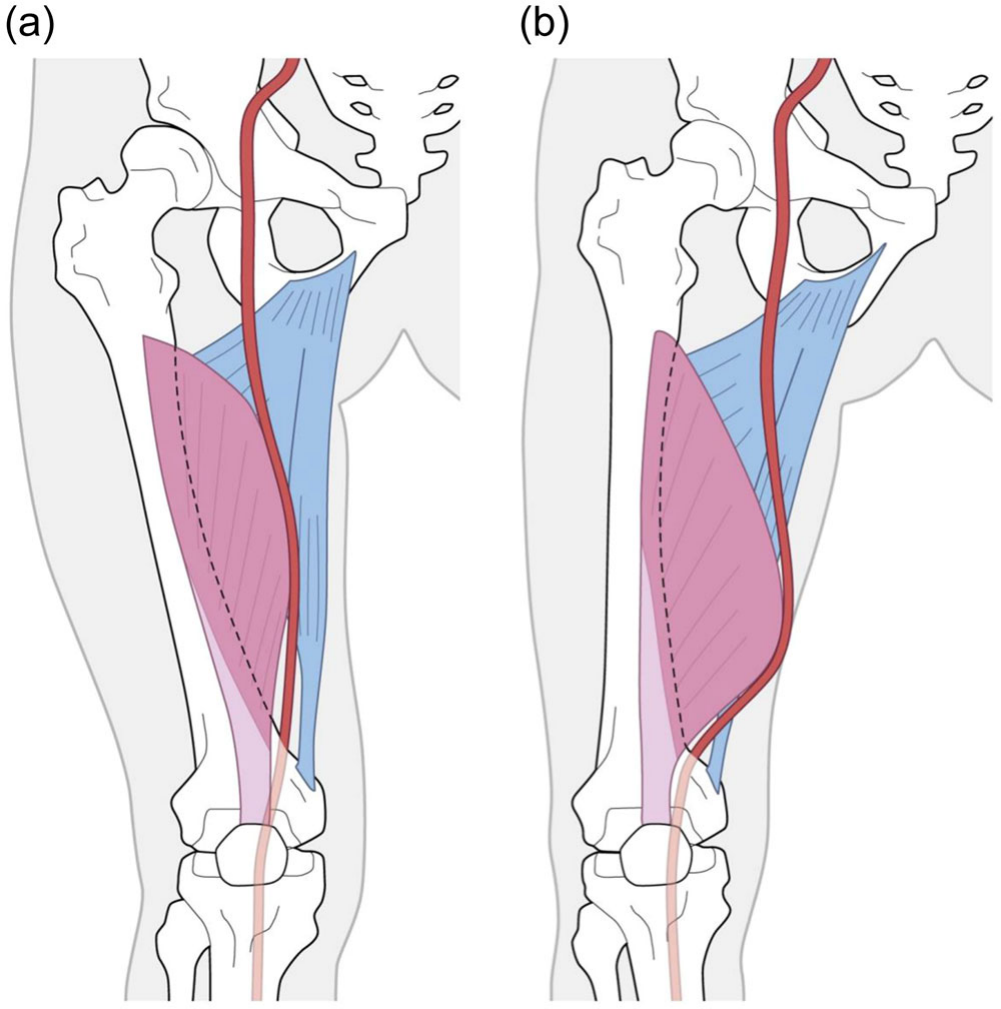
When the posterior overhang of the oblique fibres of the vastus medialis in the valgus knee is large, the image suggests that the artery may also translate posteriorly and proximally relative to the femoral axis (a) compared with that of the varus knee (b).

Considering the effect of soft tissues, the posterior wall of the adductor hiatus is less affected by changes in position due to morphological changes [9] because the insertion of the hard adductor magnus attaches to the linea aspera of the femur. However, the vastus medialis muscle, which is the lateral wall, can move depending on its shape and may affect the course of the artery. Tensho et al. [20]. reported that the vastus medialis protrudes more proximally than distally. There are reports on hypoplasia and atrophy of the valgus knee, as well as atrophy in severe varus OA [7, 18]. In this study, there was no significant difference in the mean values among the three groups, suggesting that the effects of atrophy and hypoplasia were small. In Hunter canal syndrome caused by compression in the adductor canal, there are reports that one of the causative factors is compression at the adductor hiatus due to hypertrophy of the vastus medialis muscle [1, 23]. The oblique fibres of the vastus medialis oblique muscle (vastus medialis obliquus) exhibit an inclination of approximately 50–55° with respect to the femoral axis [7, 16], and when the vastus medialis muscle is stretched out, the femoral valgus becomes more pronounced than the varus. It is thought that a vector acts in a direction that causes the artery to translate proximally and posteriorly relative to the femur, suggesting the possibility that it moves away from the distal femur (Figure 10). Based on the above, we believe that when using the subvastus approach for the valgus knee, caution is required not only for patient with short stature but also for tall patients as the vastus medialis muscle and femoral artery may run relatively distally.

**Figure 11:**
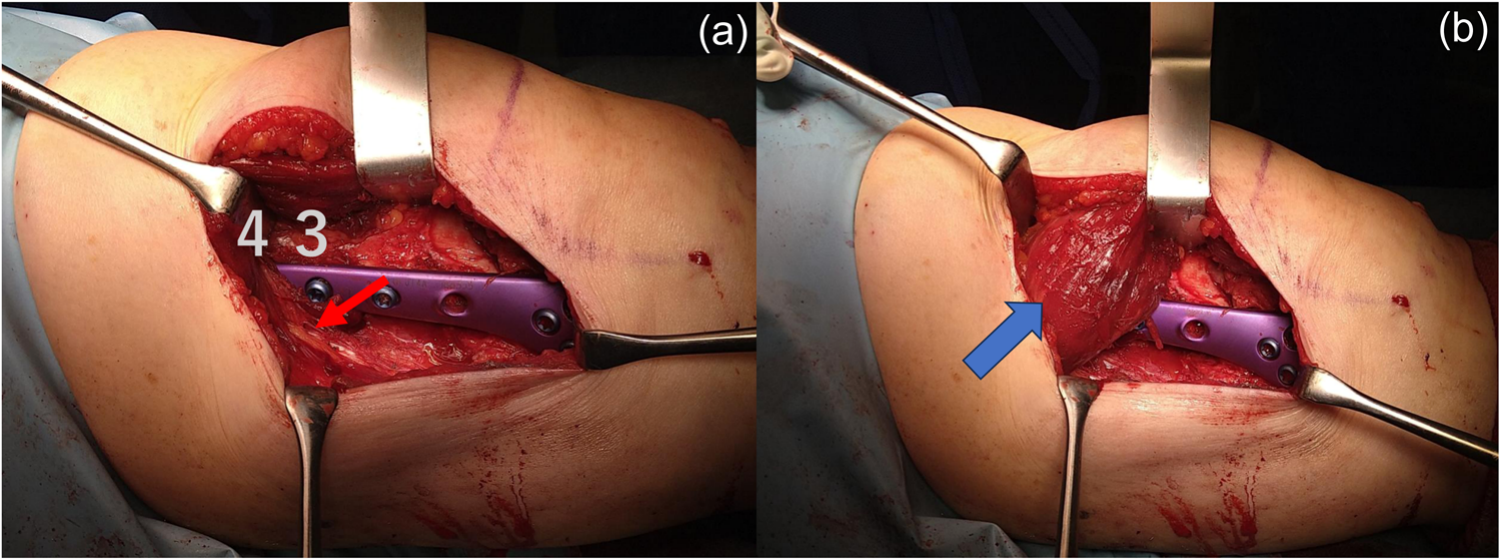
The arrow indicates the adductor magnus fascia (a). Holes 3 and 4 of the Tria Plate are usually located near these structures. Minimally invasive plate osteosynthesis and vastus medialis muscle‐splitting approaches are viable options for patients with short stature (b).

The descending genicular artery may be used to identify the danger zone of the subvastus approach during surgery. The artery first branches out from the femoral artery near the adductor hiatus and has been reported to be present in approximately 82% of cases [4]. Distal to the vastoadductor fascia, the lower border fibres of the vastus medialis oblique muscle are attached to the adductor magnus fascia, and proximal to it are the vastus adductor fascia and the adductor tendon, which may be used as a guide (Figure 11a). Since the total length of the Tris‐MDFO plate (Olympus Terumo Biomaterials) used for fixation after osteotomy was 110 mm and the mean distance from the medial epicondyle to the adductor tendon hiatus in the valgus knee in this study was 74.9 ± 6.4, the third and fourth holes are often located near the adductor hiatus. Because surgical exposure and proximal screw holes of the plate may pose a risk of damaging the femoral artery, minimally invasive plate osteosynthesis (MIPO) and vastus medialis muscle‐splitting approaches are considered viable options, especially for patients with short stature (Figure 11b).

There are notable limitations to this study, including the small number of cases, significant differences in mean age and patient height between groups, and all groups being preoperative cases that may have included a history of intra‐articular oedema, degenerative changes, and muscle atrophy. In addition, the degree of vastus medialis muscle coverage in the measurement slice cannot be said to be directly related to the vastus medialis muscle thickness, the imaging position and rotational positioning were not uniform, the bowing of the femoral shaft and presence of lateral translation were unaccounted for, the assessed images were perpendicular to the functional axis regardless of alignment, and image evaluations were performed two‐dimensionally.

## CONCLUSION

Using plain radiography and CT images, we evaluated the distance at which the popliteal artery meets the vastus medialis from the distal side at the adductor hiatus. The mean distance from the medial epicondyle was approximately 76 mm, and no significant difference was observed between alignments or differences in valgus/valgus in the slope of the distal femoral articular surface. In the valgus knee, a positive correlation was observed between the distance and degree of vastus medialis coverage, indicating that a vector may act to translate the femoral artery proximally and posteriorly relative to the femur due to the overhang of the vastus medialis muscle. Furthermore, the overhang may also affect the position of the artery from the distal femur.

The mean distance from the medial epicondyle to the adductor tendon hiatus in the valgus knees of this study was 74.9 ± 6.4, suggesting that excessive proximal dissection of the vastus medialis muscle during surgical exposure and proximal screw insertion of the plate may pose a risk of damaging the femoral artery because of their proximity to the adductor hiatus. Due to this risk, the MIPO and vastus medialis split approaches were considered viable options.

Although previous studies have reported that patient height can influence the position of the adductor hiatus, the findings in this study suggest that the morphology of the vastus medialis can also influence this position in valgus knees.

## AUTHOR CONTRIBUTIONS

Fumiyoshi Kawashima designed the study, collected the data, carried out the imaging analysis, performed statistical analysis, and drafted the manuscript. Hiroshi Takagi provided help in collecting data, performing imaging analysis, designing the study, and statistical analysis. Koji Kanzaki supervised in the design of the study and writing of the manuscript. All authors read and approved the manuscript.

## CONFLICT OF INTEREST STATEMENT

The authors declare no conflict of interest.

## ETHICS STATEMENT

Ethical approval was provided by the IRB of Showa University Fujigaoka Hospital. The patients and/or their families were informed that data from the research would be submitted for publication and gave their consent.

## Data Availability

The data that support the findings of this study are available from the corresponding author upon reasonable request.
